# A comparison of development methods used to define portion sizes in food-based dietary guidelines around the world

**DOI:** 10.3389/fnut.2025.1532926

**Published:** 2025-02-12

**Authors:** Fanny Salesse, Alison L. Eldridge, Tsz Ning Mak, Eileen R. Gibney

**Affiliations:** ^1^Institute of Food and Health, University College Dublin, Dublin, Ireland; ^2^Insight Centre for Data Analytics, University College Dublin, Dublin, Ireland; ^3^Nestlé Institute of Health Sciences, Nestlé Research, Lausanne, Switzerland; ^4^Nestlé Institute of Health Sciences Singapore Hub, Nestlé Research, Singapore, Singapore

**Keywords:** food-based dietary guidelines, portion size, dietary recommendations, linear programming, diet modeling, dietary intake

## Abstract

**Introduction:**

Food-based dietary guidelines (FBDGs) are essential public health tools for delivering dietary recommendations, and generally include guidance on portion sizes. Despite existing guidelines on developing and implementing FBDGs, there is still no consensus on best practices for their formulation. This paper compares the methodologies used by public health organizations to create FBDGs and examines how both methodology and geographical location may influence recommended portion sizes.

**Methods:**

Documents on FBDG development were obtained from the Food and Agriculture Organization online repository of FBDGs, either directly from consumer-facing FBDG or from corresponding scientific reports. Methodological details in FBDG development were extracted and categorized. Recommended portions in grams per day were extracted for 15 food categories to enable comparison across development methods and global regions.

**Results:**

FBDGs from 96 countries were accessed and translated. Of these, *n* = 83 were based on consensus/review, *n* = 15 used data-based approaches, and *n* = 30 included other minor calculations. Thirty-nine FBDGs were derived from a combination of consensus/review and another method. Of the countries providing portion size information, only one did not report its methodological approach. Comparisons of median portions sizes of food groups across methodologies showed no significant differences. Analyses across regions revealed that portion recommendations were generally consistent, with significant differences found only for one food group, namely, Fish & shellfish, where portion size recommendations were significantly higher in Europe compared to those in Latin America and the Caribbean.

**Discussion:**

Results indicate little variation in the recommendations for portion size across development methods, and for most food groups, across global regions. These findings suggest there is potential to harmonize portion size derivation in FBDGs at regional or global levels. However, further research is needed to assess whether harmonized guidance can apply to other aspects of FBDGs.

## 1 Introduction

According to the World Health Organization (WHO), unhealthy eating habits are a major risk factor for non-communicable diseases (NCDs) (1). In 2017, a systematic analysis showed that 11 million deaths and 255 million disability-adjusted life years (DALYs) were attributable to suboptimal diets (2). The current rise in obesity and outbreak of NCDs underscores the importance of dietary recommendations. As “consumers think in terms of foods rather than of nutrients” (3), various initiatives including food-based dietary guidelines (FBDGs) are used to provide nutritional information for consumers.

FBDGs, defined as “a set of healthy eating messages provided for a population” (4) represent a valuable tool in communicating dietary recommendations to populations. FBDGs aim to “improve food consumption patterns and nutritional status of individuals and populations” by promoting practical and culturally acceptable healthy diet and lifestyle habits (3). They also serve as a tool in national nutrition, health and agriculture policies. Initially introduced in the United States, they are now implemented in more than 100 countries worldwide (5). Common formats include food pyramids, which allow to visualize food groups and PS in a hierarchical manner, and food plates (e.g., “MyPlate” model in the United States), which divide a plate into sections to represent ideal proportions of different food groups (6). In addition, booklets, apps, and online resources provide detailed guidance on meal planning, portion control, and nutrient intake. Several public health authorities have published guidance on how to develop and monitor the impact of FBDGs. In 1998, the Food and Agriculture Organization of the United Nations (FAO) and the WHO published a technical report providing scientific considerations for the preparation of FBDGs (3), and the European Food Safety Authority (EFSA) released a scientific opinion on establishing FBDGs in 2010 (7). These include elements such as review of existing consumption patterns, defining the specific scope and problem for the region/country to focus the purpose, goals and targets of the guidelines, and the testing and optimisation of the developed guidelines (3, 5).

Whilst it is recognized that advice provided in FBDGs is informative, there remains no general consensus on the best practices for deriving and monitoring FBDGs. As understanding the food environment and food consumption patterns can be used to support changes in population and planetary health (8, 9), countries continue to develop or update their FBDGs to support public health targets (5). In a recent review, four commonly used components were identified for the development of FBDGs: evidence of diet-health interactions, nutrient supply, energy supply, and dietary habits. However, this report also highlighted the absence of major components such as population segmentation or the consideration of recommendations on environmental sustainability (10). In addition, Blake and colleagues looked at the quality of the evidence used to generate the guidelines and found deficiencies in the approaches used to both review the evidence and rate its quality (11). It is, however, crucial to ensure that food intake recommendations are tailored to address both global and local dietary challenges (12, 13).

FBDGs can include both qualitative and quantitative guidance. The latter, being the focus of this paper, includes the concept of portion size (PS) recommendations. PS and frequency of consumption are used to direct the overall amount of given food/food group consumers are recommended to consume. A “portion” typically refers to the suggested amount of food which an individual ingests at a single meal or eating occasion (14, 15). Frequency is the number of times the portion is recommended to be consumed in a typical day or week. Different approaches are used to derive reference PS. One approach is to base the guidance on amounts which are considered optimal for achieving desired health targets, but these can be difficult to reach in practice due to inequities in food security worldwide (16, 17). The other approach is to base portions on usual intakes, which are easier for people to understand and follow, but may not be desirable for health purposes (18), as median PS, especially for foods high in fat, salt and sugar, have increased significantly over the past decades (19–21). Usual intakes are determined using food intake data collected as part of national consumption surveys (22, 23). Impact of differing approaches used to derive PS, and their use in FBDGs has not been investigated to date. Recent studies have found similarities in the food groups recommended yet noted some discrepancies in the recommended amounts across differing FBDGs (24–26).

This research aims to review the methodologies used to develop quantitative dietary recommendations in FBDGs worldwide, with a particular focus on the use of food intake data. Our objective is to investigate the potential impact that different methodologies may have on the recommended intakes by comparing the distributions of recommended PS for each food group. A key aspect of this study is to determine the impact of the development method taking into account the local context across global regions.

## 2 Materials and methods

A comprehensive and systematic approach was taken to data collection and analysis, to ensure a clear and objective approach to the collection and handling of data within this study. Details of the methods applied, are outlined in full detail below.

### 2.1 Food-based dietary guidelines documents

The online FAO repository of FBDGs was accessed between 1 July 2023 and 12 July 2024 to obtain a list of countries with published FBDGs. All countries listed on the FAO repository were considered for inclusion in the study. An additional web search was conducted to capture latest/most updated versions of each country’s FBDGs as well as additional background documents in the gray literature, using the following keywords: “[country] food-based dietary guidelines scientific report OR scientific development.” All documents related to the listed FBDGs were accessed and screened. To read documents written in any language other than English, French or Spanish, Google Translate was applied to texts of relevant documents.

The most recent version of the FBDG documents was reviewed. For each country considered in this analysis, guidelines and recommendations aimed at the general healthy adult population were assessed. Since the analyses were restricted to adult FBDGs only, those recommendations specifically designed for infants, children, teenagers, elderly, pregnant and breastfeeding women were excluded. FBDGs were grouped by region, as presented on the FAO repository. Data, described below, was manually extracted and stored on Microsoft Excel (Microsoft Office, V.2401).

### 2.2 Categorization of FBDG development methods

The methodology used to derive quantitative recommendations was determined from the methods section of FBDGs or from their associated background or scientific report documents. The methods used were classified into three categories, as follows: 1. Scientific consensus / literature review based on groups of experts, review of published reports, or literature review of the knowledge or nutritional situation of the country, or on the associations between diet and health; 2. Minimal calculations based on different energy levels and/or certain anthropological constraints (e.g., sex differences); and 3. Data-based approaches using data modeling that included a combination of constraints for energy, nutrients, and foods or food groups applied to a suitable data set (e.g., linear programming). Upon review of the documentation, we noted that several countries applied more than one method (e.g., scientific consensus and data-based approach). Therefore, all methodological approaches used were captured, allowing multiple methods to be listed for each FBDG. For the purpose of this analysis, when more than one methodological approach was applied, i.e., consensus/review plus either calculations or data-based approaches, then the FBDG was classified according to the additional method, with the aim to compare the recommendations between FBDGs using consensus/review only and FBDGs using calculations and statistical approaches. When no information was provided regarding the development of the FBDG, it was classified as “Not specified.” Some FBDGs reported following specific methodologies outlined in regional guidelines; in this case, the methodologies were more often detailed in these reports rather than in the national FBDGs, and the detailed information was collected from the referenced documents. When a data set was used, details pertaining to its composition, including cohort representativeness, data collection methodology, and other relevant characteristics, were obtained from external documentation sources. These included peer-reviewed articles or supplementary information provided by the original dataset creators.

### 2.3 Portion sizes

A standardized approach was applied to determine the PS of each food group included in our analyses. When PS was provided as gram amounts at an overall food group level, no conversion was necessary. If PS for different foods were given within a food group, the average recommended portion (g) of the individual food values was calculated. In the case of PS recommendations given in other units (e.g., cup, food item, tablespoon) these were converted to a gram equivalent using two sources: the Food Portion Sizes Book (version 3) (27) and the USDA’s Food and Nutrient Database for Dietary Studies (FNDDS) 2017–2018 (28). When both sources provided a gram equivalent for the food, an average was computed. When only one had an equivalent, then its value was used. A visual aid tool (29) was used to convert recommendations provided in other units (e.g., hand, palm, plate). If the document contained recommendations for different daily energy levels based on physical activity, the values corresponding to a medium activity level were considered. When a range of values was provided instead of a single amount, the mid-point of the range was reported. In addition, specific rules were applied for each food groups, which are detailed in Supplementary Table 4.

For each FBDG, portions were manually converted into gram amounts for each food and food group. Quality checks were conducted by the lead author and PS values were reviewed by all team members. Outliers were identified and values were discussed within the research team. Three values were excluded from the calculation, as they were deemed implausible from a dietary intake perspective (e.g., in the Mexican FBDG, the recommendation for vegetables included a “1.5 raw cabbage” which when converted to a gram amount represented a PS of 1,050 g (700 g per cabbage × 1.5). Values for global regions were obtained by calculating medians and the interquartile ranges (IQR) or each food group.

Data for the following food groups was extracted from the FBDGs as described above: Fresh fruits; Vegetables (unspecified); Vegetables (excluding green/leafy); Vegetables (green/leafy only); Cooked cereals/grains; Bread; Potatoes, starchy fruits and vegetables; Milk / plant-based alternatives; Yogurts and fermented dairy; Cheese; Meat; Fish & shellfish; Eggs; Pulses; Nuts & seeds.

### 2.4 Comparative analyses

Kruskal–Wallis tests were applied to compare the distributions of recommended PS of food groups across regions and across methodological approaches (30). Wilcoxon Rank-Sum tests (31) were applied to compare the distributions between data-based approaches and other approaches combined. For both tests, *p*-values were adjusted for False Discovery Rate (FDR) using Benjamini-Hochberg procedure (32). Post-hoc analyses were performed when the *p*-value was below 0.05, consisting of a Dunn-s test with Bonferroni correction for multiple testing (33). Comparisons were performed across regions, across development methods and between data-based approaches and other methods, specifically to examine the potential impact of using survey data. Analyses were performed on RStudio version 4.2.2.

## 3 Results

### 3.1 Included food-based dietary guidelines

At the time of data extraction, 100 countries were listed on FAO repository of FBDGs. Of these, three FBDGs were excluded as the documentation needed was not accessible online (Iran, Nepal, United Arab Emirates). A fourth FBDG was also excluded, because its recommendations targeted only children (Cambodia). Therefore *n* = 96 countries were included in the analysis: *n* = 2 in North America, *n* = 11 in Africa, *n* = 34 in Europe, *n* = 16 in Asia, *n* = 29 in Latin America and the Caribbean (LAC), and *n* = 4 in the Near East. Supplementary Table 1 lists the FBDGs included from each region, the access link to their consumer material from which PS were extracted, as well as the access link to the material reporting the development methodology when it was provided on a separate document.

### 3.2 Methodological approaches applied to derive food intake guidance in FBDGs

Table 1 summarizes the methodologies used to determine dietary recommendations in FBDGs, by FAO region. The specific approach used by each country are provided in Supplementary Table 2. The majority of countries (*n* = 83) mentioned either the formation of a group of experts, a review of the nutritional status of the population, or an evaluation of the associations between diet and health in their guidelines. Of these, *n* = 39 additionally conducted calculations, either minimal or data based. Overall, about a third (*n* = 30) of the 96 FBDGs analyzed included minimal calculations. However, relatively few countries included data-based approaches in their dietary guidelines, with only *n* = 15 of them describing a programming method. Seven out of 96 countries did not specify the method used. Among these, six did not include any PS recommendations (see Supplementary Table 2). The remaining country, Slovenia, provided PS recommendations but did not report the methodological approach used to develop them (“Not specified”). As a result, Slovenia was excluded from the statistical comparisons across methods.

**TABLE 1:** Methodological approaches applied to determine dietary recommendations in FBDGs by FAO region.

Region	*n* FBDGs	Methodological approaches applied^1^			
		Literature/evidence review, scientific consensus	Minimal calculations	Data-based approaches	Not specified
North America	2	2	0	1	0
Africa	11	9	2	4	1
Europe	34	31	9	5	2
Asia and the Pacific	16	15	6	3	1
Latin America and the Caribbean	29	22	12	1	3
Near East	4	4	1	1	0
Global	96	83	30	15	7

^1^Each FBDG may be based on more than one method.

For the countries who reported using a data-based approach, Table 2 provides the main characteristics of the dietary data and variables used within the analysis for the derivation of recommended intakes. While different titles were used to describe the process (e.g., “programming,” “optimisation,” “modeling”), data-based approaches generally involved applying a set of diverse food group and nutrient constraints to meet dietary needs. These procedures often utilize dietary intake data and consider local eating habits to ensure that the recommendations align with typical consumption patterns. However, when considering the data reported to be used only *n* = 8 FBDGs mention using a nationally representative dataset as an input in their model (Australia, Denmark, France, Germany, the Netherlands, Oman, United Kingdom, United States). All reported datasets used were national food consumption surveys, except for Oman where the data used was a household expenditure and income survey. FBDGs for Estonia, Finland, Iceland, Latvia, Norway and Sweden were adapted from the Nordic Nutrition Recommendations (34), and Dominica, Grenada, Saint Lucia and Saint Vincent and the Grenadines were developed after the FAO Manual from the English-speaking Caribbean (35).

**TABLE 2:** Characteristics of the dietary data used for the derivation of recommended intakes in FBDGs.

Development method	Country	Survey/data used	Years of data collection	Food intake assessment method	Nationally representative (yes/no)	References
Linear programming	Benin	Different cross-sectional surveys	2005–2006	2 to 3 days 24-h recall	No	(77)
	United Kingdom	National Diet and Nutrition Survey (NDNS)	2008–2011	3 days 24-h recall	Yes	(78)
Diet modeling	Ethiopia	Cross-sectional National Food Consumption Survey (NFCS/EFCS)	2011	1 day 24-h recall	No	(79, 80)
	Zambia	US and West African food composition tables for nutrient analysis, Zambia’s food consumption data	NS	n/a	No	(81)
	Ghana	Different surveys	NS	n/a	No	(82)
	Sri Lanka	Survey conducted by Wayamba university: sample of rural, urban and estate populations	2015–2017	1 day 24-h recall	No	(83)
Food pattern modeling	United States	National Health and Nutrition Examination Survey (NHANES)	2013–2016	2 days 24-h recall	Yes	(84)
	Oman	Omani household expenditure and income survey (OHEIS)	1999–2000	n/a	Yes	(85, 86)
Food modeling	Australia	National Nutrition Survey (NNS)	1995	1 day 24-h recall	Yes	(87)
Model calculations	Denmark	Danish National Survey of Diet and Physical Activity (DANSDA)	2011–2013	7 days food diary	Yes	(88)
Optimisation	France	Etude Individuelle Nationale des Consommations Alimentaires (INCA2)	2005–2007	7 days food diary	Yes	(89, 90)
	Netherlands	Dutch National Food Consumption Survey (VCP)	2007–2010	2 days 24-h recall	Yes	(91–93)
	Germany	German National Nutrition Survey II (NVS II)	2005–2007	2 days 24-h recall	Yes	(94)
Not named (mentions “model”)	Thailand	Sample of 20 households, and five sets of secondary data from the Institute of Nutrition, Mahidol University-INMU (unpublished data)	NS	n/a	No	(95, 96)
Not named	Costa Rica	Latin American Study of Nutrition and Health (ELANS), and home measurements and food composition database	2015	2 days 24-h recall	Yes	(97)

NS, not specified; n/a, not applicable.

### 3.3 Comparison of portion size recommendations across regions and methods

Not all countries included PS recommendations in their FBDGs, with *n* = 26 countries not providing PS recommendations for any of the 15 food categories examined. Thus, the PS comparisons within the work presented here were based on FBDGs from 70 countries organized into six global regions (Table 3). A comparison of recommended PS across the six global regions is presented in Table 3, with a global median included for reference. Significant variation was observed for Bread, Meat, and Fish & shellfish, as indicated by *p*-values below 0.05. However, after adjusting for FDR, only the PS recommendations for Fish & shellfish remained significantly different across regions (*p* = 0.02). Specifically, Europe had higher recommended PS for Fish & shellfish compared to Latin America & the Caribbean (LAC), with a Bonferroni corrected *p*-value of 0.005.

**TABLE 3:** Distribution of portion size recommendations in FBDGs, per region.

Food	Statistic	Global	Africa	Asia and the Pacific	Europe	Latin America and the Caribbean	Near East	North America	p^1^	Adj p^2^	*Post hoc* analysis adj p^3^
Fresh fruits	*N*	66	7	9	28	17	4	1			
	Median	127.6	130.6	124.0	119.5	134.5	138.7	153.5	0.490	0.628	n/a^5^
	IQR^4^	41.2	19.0	34.2	50.0	16.3	33.0	0.0			
Vegetables–unspecified	*N*	39	3.0	5.0	21.0	10.0	0.0	0.0			
	Median	100.0	80.0	100.0	100.0	99.6	n/a	n/a	0.503	0.628	n/a
	IQR	30.4	43.3	0.4	40.0	11.6	n/a	n/a			
Vegetables–excl. green/leafy	*N*	27	5	5	6	6	4	1			
	Median	100.4	86.7	81.6	118.8	100.4	119.4	128.3	0.511	0.628	n/a
	IQR	56.9	50.7	11.3	62.3	17.8	45.6	0.0			
Vegetables—green/leafy	*N*	26	5.0	5.0	7.0	4.0	4.0	1.0			
	Median	70.0	50.0	47.3	80.0	86.8	73.8	54.0	0.708	0.708	n/a
	IQR	46.6	63.3	20.0	38.1	46.8	33.0	0.0			
Cooked cereals/grains (rice, pasta, …)	*N*	49	4	10	21	10	3	1			
	Median	90.0	142.3	98.8	85.0	90.0	78.2	74.5	0.544	0.628	n/a
	IQR	58.8	40.4	59.6	65.5	24.3	4.3	0.0			
Bread	*N*	48	6	7	20	11	3	1			
	Median	41.1	78.8	50.0	47.8	39.6	26.9	28.4	0.032	0.160	n/a
	IQR	23.7	93.0	71.0	23.0	10.0	5.2	0.0			
Potatoes, starchy fruits and vegetables	*N*	39	3	7	17	12	0	0			
	Median	137.5	140.0	100.0	138.0	115.0	n/a	n/a	0.115	0.215	n/a
	IQR	60.3	19.0	55.9	80.3	58.3	n/a	n/a			
Milk / plant-based alternatives	*N*	54	4	10	25	11	3	1			
	Median	222.0	222.5	200.0	222.0	222.0	244.0	244.0	0.657	0.704	n/a
	IQR	44.0	46.3	103.9	50.0	32.5	2.0	0.0			
Yogurts and fermented dairy	*N*	46	4	6	21	11	3	1			
	Median	181.8	162.5	124.0	170.0	188.5	245.0	245.0	0.081	0.203	n/a
	IQR	86.0	112.5	77.0	65.0	58.3	6.5	0.0			
Cheese	*N*	52	4	6	25	13	3	1			
	Median	39.0	27.5	40.0	50.0	30.0	52.5	49.6	0.368	0.614	n/a
	IQR	39.7	11.3	44.6	47.5	2.5	7.5	0.0			
Meat	*N*	53	6	9	20	14	3	1			
	Median	75.0	77.7	72.5	92.5	66.7	30.0	28.4	0.015	0.115	n/a
	IQR	40.0	3.9	33.3	29.9	28.4	22.5	0.0			
Fish & shellfish	*N*	51	6	9	23	9	3	1			
	Median	90.0	98.1	70.6	120.0^a^	38.1^a^	75.0	28.4	0.001	0.021	0.005^a^
	IQR	75.8	31.1	55.0	50.0	42.9	30.0	0.0			
Eggs	*N*	47	6	8	18	12	2	1			
	Median	50.0	65.0	50.0	52.5	50.0	50.0	50.0	0.053	0.183	n/a
	IQR	9.0	38.3	23.3	40.0	0.0	0.0	0.0			
Pulses	*N*	53	7	9	20	13	3	1			
	Median	92.5	95.0	100.0	127.5	80.3	90.7	45.8	0.115	0.215	n/a
	IQR	70.0	27.9	105.0	95.0	64.4	23.0	0.0			
Nuts & seeds	*N*	35	6	6	13	7	2	1			
	Median	23.5	20.8	30.0	25.0	13.3	15.0	14.2	0.068	0.183	n/a
	IQR	15.0	13.5	11.3	5.0	4.7	0.0	0.0			

^1^*p*-value for Kruskal–Wallis test.

^2^Adjusted *p*-value for Kruskal–Wallis test (adjustment for False Discovery Rate—Benjamini Hochberg).

^3^Adjusted *p*-value for Dunn’s test adjusted with Bonferroni correction–run if adjusted *p*-value for KW test was below significance level of 0.05.

^4^Interquartile range.

^5^Not applicable.

^a^Indicates significant difference between groups, from *post-hoc* analysis (corresponding *p* = 0.005).

Table 4 provides the comparison of PS recommendations in FBDGs across the three different methodological approaches considered. While unadjusted *p*-values showed statistically significant differences for the Meat, Fish & shellfish and Pulses food groups, none remained significant after adjusting for FDR. Therefore, this analysis did not identify any association between the approach used in a FBDG and its respective recommended PS.

**TABLE 4:** Distribution of portion size recommendations in FBDGs, per development method.

Food	Statistic	Global	Consensus/ review only	Data-based	Minor calculations	p^1^	Adj p^2^	*Post hoc* analysis adj p^3^
Fresh fruits	*N*	65	26	14	25			
	Median	125.6	125.1	122.3	130.5	0.637	0.735	n/a^5^
	IQR^4^	40.6	37.9	46.3	35.0			
Vegetables–unspecified	*N*	39	16	8	15			
	Median	100.0	100.0	80.0	100.0	0.063	0.236	n/a
	IQR	30.4	52.3	19.5	17.7			
Vegetables–excl. green/leafy	*N*	26	9	6	11			
	Median	98.6	96.8	105.6	100.4	0.987	0.987	n/a
	IQR	53.2	24.3	44.5	62.7			
Vegetables—green/ leafy	*N*	25	10	6	9			
	Median	75.0	83.3	52.0	80.0	0.282	0.704	n/a
	IQR	44.5	40.1	6.0	29.5			
Cooked cereals grains (rice, pasta, …)	*N*	48	16	9	23			
	Median	90.0	95.0	78.2	90.0	0.540	0.735	n/a
	IQR	59.3	65.2	61.5	42.0			
Bread	*N*	47	16	10	21			n/a
	Median	42.3	37.0	41.9	50.0	0.523	0.735	0.735
	IQR	24.4	15.2	35.5	25.5			
Potatoes, starchy fruits and vegetables	*N*	38	13	6	19			
	Median	136.3	138.0	137.8	125.0	0.686	0.735	n/a
	IQR	62.6	81.7	28.6	64.2			
Milk / plant-based alternatives	*N*	53	19	11	23			
	Median	222.0	222.0	244.0	205.0	0.338	0.725	n/a
	IQR	44.0	47.0	47.5	67.0			
Yogurts and fermented dairy	*N*	45	15	10	20			
	Median	188.5	188.5	200.0	169.3	0.504	0.735	n/a
	IQR	88.0	41.5	113.8	82.1			
Cheese	*N*	51	19	12	20			
	Median	38.0	41.3	27.5	35.0	0.137	0.412	n/a
	IQR	39.3	40.5	29.7	40.3			
Meat	*N*	52	19	11	22			
	Median	75.0	82.0	75.0	64.6	0.019	0.168	n/a
	IQR	41.3	23.8	30.7	39.3			
Fish & shellfish	*N*	50	19	12	19			
	Median	90.0	115.0	100.0	48.3	0.022	0.182	n/a
	IQR	77.9	71.8	42.6	56.9			
Eggs	*N*	46	13	11	22			
	Median	50.0	50.0	50.0	50.0	0.439	0.735	n/a
	IQR	9.5	0.0	35.5	5.0			
Pulses	*N*	52	18	11	23			
	Median	92.3	122.5	92.0	80.0	0.049	0.236	n/a
	IQR	66.3	88.6	24.2	73.9			
Nuts & seeds	*N*	35	10	10	15			
	Median	23.5	25.9	20.8	17.3	0.667	0.735	n/a
	IQR	15.0	10.6	10.0	14.4			

^1^*p*-value for Kruskal–Wallis test.

^2^Adjusted *p*-value for Kruskal–Wallis test (adjustment for False Discovery Rate—Benjamini Hochberg).

^3^Adjusted *p*-value for Dunn’s test adjusted with Bonferroni correction–run if adjusted *p*-value for KW test was below significance level of 0.05.

^4^Interquartile range.

^5^Not applicable.

**FIGURE 1:**
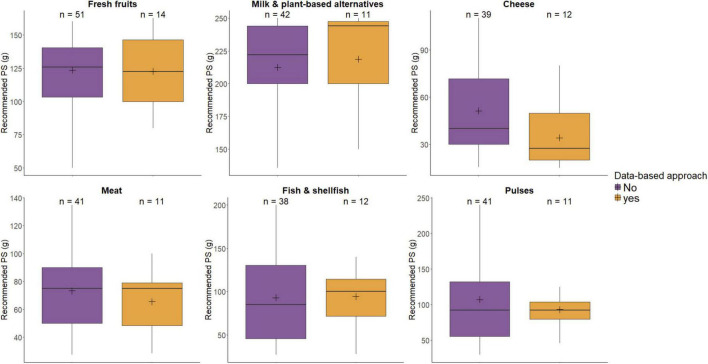
Distribution of PS recommendations in FBDGs, data-based versus other methodological approaches.

Figure 1 illustrates the comparison of PS recommendations in FBDGs when methodological approaches were grouped by data-based approaches versus those that used other methods (Consensus/review and Minor calculations), for selected food groups. The full data for all 15 food groups and Wilcoxon Rank-Sum test can be found in Supplementary Table 3. No significant differences were observed between the PS recommendations derived via data-based approaches and those derived via other methods.

## 4 Discussion

This study identified three primary methodological approaches that were used to develop portions sizes within FBDGs in several countries around the world: consensus/literature review, minor calculations, and data-driven approach. We sought to characterize these to examine the impact of the methodologies and geographical regions on recommended PS of key food groups. Our analysis showed that many FBDGs were based solely on existing scientific evidence in the development of their FBDG either by conducting literature reviews or forming expert opinions. Only *n* = 15 relied on the use of data, of which even fewer completed detailed dietary modeling using relevant national food consumptions surveys. When we considered the impact on PS, we found the region rather than methodological approach had a greater influence.

While comparisons across methods were considered within this paper, it is important to remember that each approach has merit and is selected based on available data, resources and specific context being considered. Each has its own strengths and weaknesses. For example, it is well known that consensus approaches can draw on a collective knowledge of experts in any given field, allowing for the inclusion of insights that may not be explicitly detailed in existing literature (36, 37). This is also the case for addressing challenges such as planning and developing nutrition guidance (38, 39). However, caution in the use of this approach is also warranted. In their analysis of 32 FBDGs, Blake et al. (11) reported that most countries relied on a consensus-based approach to formulate their recommendations, which is similar to the findings presented here. However, they noted that this approach was often applied without grading the strength of such recommendations, and very few countries conducted a formal systematic review (11, 40). In the present study, we focused on the impact of using a data modeling approach versus not, and combined methodologies reported as consensus and review, and also found that the majority of FBDGs used this approach. Looking at this in more detail, only a very small number of countries conducted a systematic review, relying mostly on scientific consensus of informed experts. This approach has been open to criticism in more recent years, due to potential bias and conflicts of interest (41, 42). This aspect was not examined in the current study but is worthy of further investigation.

Differing from previous studies, our work specifically examined the use of data in the development of guidelines. Fewer than half of the FBDGs combined consensus or review with other approaches, which varied from minor calculations to complex dietary modeling. A blended approach aims to ensure that guidelines are based on high-quality evidence, while remaining practical and applicable for the target population (10). For instance, guidelines from the US Dietary Guidelines Advisory Committee (DGAC) utilized both consensus from experts and data-driven insights, creating a comprehensive framework that encompasses various viewpoints and research findings. Other examples for the use of combined methods are Germany (scientific consensus/review and data-based approaches) and Cuba (scientific consensus/review and minor calculations).

A key finding from this work is that there are currently limited data-driven FBDGs, and there is a need to increase the availability and use of data in the development of such recommendations. Supporting and informing future developments of FBDGs, several European funded initiatives, such as Plan’EAT (43) and FEAST (44), are developing harmonized strategies for FBDG development, incorporating sustainability as a core element. We have also more recently seen regional collaborations, such as the Nordic Nutrition Recommendations (NNR) or the EAT Lancet diet, which demonstrate the potential of unified frameworks that can be adapted locally. Additionally, platforms that facilitate data sharing such as EFSA and WHO GIFT, will play a crucial role in supporting these efforts by providing local data for contextualisation of regional collaboration or unified frameworks (45), thus promoting consistency in public health practices across regions.

A major challenge in deriving FBDGs from typical intakes is the scarcity of high-quality food consumption data, especially nationally representative food consumption surveys (46, 47). We found that only 8 of the 96 included FBDGs used such surveys. Many countries have limited datasets available, as they require substantial resources to collect and analyze (48). Furthermore, the scope, size and detail of the existing datasets can vary significantly, not always representing the broader population accurately, or its dietary habits throughout the year, addressing seasonal variation. In the FBDGs of Ethiopia and Sri Lanka for example, the analyses were based on a 24-h recall limited to one day, from which usual intakes cannot be precisely derived (49). In fact, lack of broad applicability of the data used was noted in the Ethiopian documents, where the authors reported that intakes might have been significantly influenced by the seasonality of the survey. Access to food consumption data is also not equal across global regions (50). The lack of dietary data, particularly in low and middle-income countries, is a widely known issue that has been reported previously (51–53). In this context, public health measures are needed to support countries in overcoming their difficulties to assess the nutritional status of their population (48, 54). Efforts to harmonize food data across Europe and beyond, such as those led by the EFSA (55) and initiatives like the Food Nutrition Security (FNS) Cloud (56), could improve the accessibility of these tools, and subsequent data collection and availability. Enhanced data standardization would help streamline the process of developing and updating these guidelines across regions (57, 58). While the FAO and WHO advocate for a review of food consumption patterns as one of the steps in developing FBDGs, they note that different types of data that can be utilized, offering different options depending on the local data availability (3).

Whilst we recognize that incorporating data in developing policies and public health tools such as FBDGs is valuable, the use of dietary intake data comes with certain limitations which should also be considered. Diet modeling is a flexible and robust approach to translate nutrient recommendations into realistic food choices, but it is very sensitive to the quality of the data used, which can be varied and influenced by the survey duration (number of days on which the estimates are based) (48), the data collection methodology used (e.g., food frequency questionnaire, dietary record) (59) and under-reporting, which occurs across all self-reported food intake data (60, 61).

Regardless of the approach used in the FBDG development process, our study did not reveal significant differences in recommended PS. Nevertheless, certain methodological limitations could affect these findings. For example, some specific details from the FBDG documents were possibly lost in translation. Additionally, relying on two specific data resources for converting recommended food amounts to grams, when needed, may have introduced some bias in PS estimation. However, the use of these documents, and any assumptions made are clearly articulated in the current work. It is also important to highlight that this observation was based on an analysis where only 15 out of 96 of the sources employed data-based approaches, which may limit the generalizability of the findings. As more nutritional surveys are initiated (45, 46), the use of data-driven methods is likely to increase, potentially strengthening the evidence base for future FBDGs. Consequently, the findings of this study may need to be revisited as the availability of data grows, alongside the adoption of novel statistical approaches involving metabolomics, machine learning, meal pattern analysis, and others (62–65). Furthermore, along with the lack of differences seen across methods, comparisons across global regions revealed no significant differences in the recommended PS, except for Fish & shellfish, between European and Latin American FBDGs. A possible reason for the significantly lower PS recommendation for Fish & shellfish in Latin America and the Caribbean compared to that in Europe could be the alignment of guidance to local dietary habits or broader and more complex issues such as cost, and availability. Indeed, other studies have shown that fish consumption is low in Latin American countries, with lower socio-economic groups consuming notably less of this food category (66).

While the overall consistency across regions might reflect a certain degree of consensus, the wide range of observed PS values suggests that underlying drivers could influence these recommendations in ways not fully captured in the present analysis. The work presented here focused specifically on PS, which may not have varied, but other facets may have, such as the consideration of sustainability or affordability of the diet, which are mentioned in many guidelines (12). Indeed, food consumption relies on many factors including ethnography, agronomic context, and economics (67–69). As these fall beyond the scope of our analysis and were not addressed in this paper, further investigation is necessary to ensure that no critical factors have been overlooked in identifying potential additional sources of variation. In particular, the incorporation of sustainability messages in FBDGs may increasingly influence recommended amounts. For instance, by recommending small PS of meat, certain countries (e.g., Germany, Costa Rica) already encourage healthy eating while advancing environmental goals.

Moreover, the consistency of PS values identified in the current analysis does demonstrate the potential of extending guidelines to a regional or even a global level. At the European scale for example, the authors of a recent analysis of PS recommendations in European FBDGs concluded that defining standardized portions could promote healthy eating programmes common to many countries, while respecting local dietary habits, and would also facilitate the communication of nutritional information by referring to quantities of a food product actually consumed, rather than to 100 g or ml (25). Additionally, Yamoah et al. (70) looked at trends in PS consumption across 24 world countries and concluded that standardization of strategies for food portions are relevant. A common concept could in fact serve as a framework for the creation of national FBDGs and could be adapted to specific local conditions by suggesting locally relevant food choices within the common food groups (10). As noted, some dietary guidelines are taking this approach, being developed at a regional level, including Nordic Nutrition Recommendations (71), which suggests that such consensus does lend itself to broad over-arching recommendations within regions.

To our knowledge, this study is the first to examine the extent to which data are used to derive recommendations within FBDG, and to compare recommended PS across potential key drivers of variation (i.e., global regions and development methods). FBDGs are often created using different sources and types of information. How these data sources/types are categorized are subjective, and the categorization used in this paper (consensus/ review and data-based approaches) may omit the fact that consensus opinions can be based on a certain knowledge of data which was not specifically listed. Furthermore, while the statistical analysis did not report differences in recommended PS across methods and across regions for most studied food groups, the large IQRs observed suggest variations in the guidance provided to consumers, which may lead to different nutritional outcomes. For example, the global IQR for portions of Pulses was of 70 g across regions, and that of Fresh fruits was 41 g. Such ranges can, respectively, correspond to differences of 17 g of proteins for a portion of lentils and 24 mg of vitamin C for a portion of orange (72), therefore considerably impacting nutrient intakes.

Coordinated approaches in the development of PS, associated with FBDG recommendations, would assist regional and national groups in developing PS recommendations in a systematic manner, avoiding duplication of effort, and reducing development costs (10). Harmonizing PS recommendations could facilitate the development of FBDGs, ensuring consistency across countries and ultimately contributing to improved public health outcomes globally. To achieve this, understanding whether various recommended portions within the observed ranges derived through different methodologies result in varying levels of adherence is crucial. Indeed, recent research has shown that many individuals are falling short of their national recommendations, particularly for fruits, vegetables, and starchy foods, but overconsume discretionary foods (73, 74). Modifying PS recommendations within FBDGs could therefore have limited impact, as several barriers to PS control have been identified. These include social and psychological factors, and childhood habits which may be difficult to overcome (75). Population approaches to reduce PS would indeed require a change in the food environment in order to have a significant impact on populations’ intakes (76), therefore the dietary habits of the target populations need to be considered when deriving recommended amounts (10). While this study focused on methods to develop FBDGs, investigating procedures to monitor their effectiveness and people’s adherence to established recommendations could also inform effective strategies for future updates.

While the development of FBDGs is led by policymakers, it may also be pertinent to consider some consultation with other stakeholders including academic researchers, consumers, public health bodies, as well as other stakeholders. This wide and encompassing approach could ensure mutual involvement in adopting healthy and appropriate PS where relevant. Establishing healthy and contextually appropriate PS is a key step in guiding, informing, and supporting consumer choices effectively.

In conclusion, data-based approaches can enhance literature reviews/scientific consensus to strengthen the rationale and assess the potential impact on dietary intakes from FBDG recommendations. In addition, policy makers should aim to harmonize PS derivation methods globally, reaching a balance between optimal and usual intakes (18). Such a concept is possible but requires investment in development and implementation; this could serve as a starting point for the derivation of the national FBDGs and be adapted to the specific local circumstances (10).

## Data Availability

The original contributions presented in this study are included in this article/Supplementary material, further inquiries can be directed to the corresponding author.
